# Data on learners emotional states, mental responses and fuzzy learning flows during interaction with learning environment

**DOI:** 10.1016/j.dib.2019.104378

**Published:** 2019-08-12

**Authors:** Mohammed Megahed, Ahmed Asad, Ammar Mohammed

**Affiliations:** Cairo University, Faculty of Graduate Studies for Statistical Research, Egypt

**Keywords:** Fuzzy, Deep learning, Facial expressions, Learners, Learning environment, Emotional states

## Abstract

The emotional state of the learner is an important factor that must be taken into consideration during evaluating learning process and managing learning flows in computer based learning environments. This factor has a significant impact on the process of interaction between the learner and the learning environment. Enriching this type of interaction make the learning flow more dynamic based on emotional and mental responses of the learners. This approach can manage various learning flows based on learner's capabilities which lead to enhance the learning process outcome. This article provides data on learners' emotional states during their interaction with learning environment and other data that describe their learning activities and learning flows. The learning activities data is a combination of data that represents summary of learners' emotional states and data that represents the mental responses per learning session. All of emotional states data and mental responses data are used to provide the next learning level for each learner using fuzzy rules. The datasets are hosted in the Mendeley Dataset Repository (Megahed, 2019).

**Table:** Specifications table

Subject area	*Human Computer Interaction*
More specific subject area	*Learners Emotional and Mental Interaction*
Type of data	*Tables, Figures*
How data was acquired	*Direct experiments on a group consisted of 12 learners*
Data format	*Raw and analyzed*
Experimental factors	*To examine how the integration between emotional states as emotional responses and mental responses of the learner during the interaction with the learning environment moderate the learning flow using fuzzy system*
Experimental features	*A set of features that is categorized into two types of features. The first type represents seven features that identify different classes of facial expressions which determine learner emotional response. The second type represents three features which identify the mental response of the learner. All these ten features are entered into a fuzzy system to generate a new feature representing the next learning level that draws the learning path of the learner.*
Data source location	*Data collected using real time interaction between learners and learning environment based on real time facial expressions detection to capture learners' emotional states and fuzzy system to detect the desired learning levels based on both emotional and mental responses of the learners during the interaction with the learning environment.*
Data accessibility	Public repository on Mendeley Data: https://doi.org/10.17632/y4dyvkrp2r.2 Direct link: https://data.mendeley.com/datasets/y4dyvkrp2r/2

**Table:** 

**Value of the data** • The data can be used to provide decision makers in education domain with information about learning achievement of all learners who participate in the learning activities • The data can be used to study the deviation of learners' emotions during the learning activities, because it describes the emotional states of learners during the interaction with learning environment. The emotional state is represented by seven classes of facial expressions • The data can be used to explain the learning flows of each learner using fuzzy logic based on learners emotional and mental responses • The data can be used to train machine learning models to predict the next learning level of the learner based on his emotional and mental responses while interacting with learning environment • The data can be used to train machine learning models to cluster the learners based on their emotions while interacting with learning environments

## 1 Data

Our collected data are categorized into two types of data. First type of data describes the features of learners’ emotional states as emotional responses, while the other type of data describes the combination of data that represents summary of learners' emotional states and mental responses per learning session.

The emotional response of the learner is represented by seven classes of expressions which are angry, disgust, fear, happy, neutral, sad, and surprise. While the mental response is represented by other factors which are managed by the learning environment; these factors are learner id, learning activity id, answer validity ratio, test elapsed time, current learning level, and desired learning level.

The *answer validity ratio* describes the validation degree of learner's answer to all the test questions. The *test elapsed time* describes the time that spent by the learner to finish a provided test. The *current learning level* represents the learner's learning level at the start of the learning activity. The *desired learning level* represents a value that is generated by a fuzzy system to redirect the learner to the next learning level based on the level and degree of emotional and mental responses of the learner.

The collected data are divided into three datasets [1]. The first data set is learners’ emotional states, the second dataset is learning activities dataset using mean, and the third dataset is learning activities dataset using median. These datasets are explained as follows:

• Learners' emotional states dataset describes the learners' emotional states during their interaction with the learning environment. The description of this dataset is explained in Table 1

**Table 1:** Description of emotional states dataset.

Number of features	9 numerical features represent learner Id, learning activity id, and seven classes of ordered facial expressions which are representing angry, disgust, fear, happy, neutral, sad, and surprise. All of these features are significant due to their importance in describing the deviation of learners' emotional states during the learning activities except both learner id and learning activity id features.
Number of instances	1735
• Learning activities dataset using mean describes the summarized emotional states using mean and its impact on the value of the next learning level of the learner which is generated by a fuzzy system. The description of this dataset is explained in Table 2. Using this dataset we provided visualization for the below:
  a. Aggregated emotional states of five learners across four learning activities for each learner as shown in Fig. 1

**Fig. 1:**
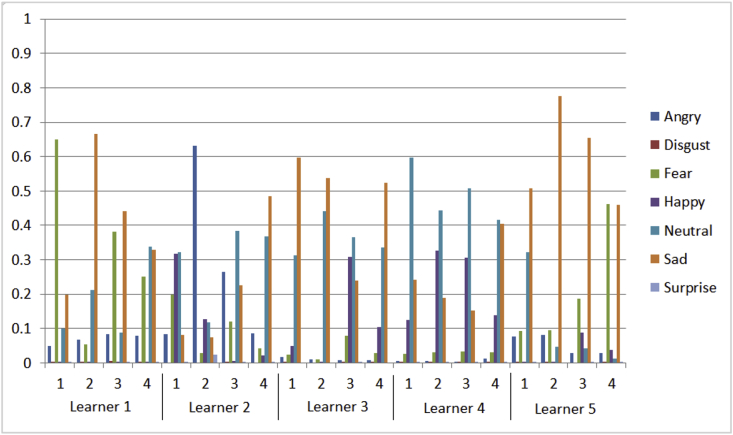
Mean of emotional states of five learners across four learning activities.
  b. Learning paths for five learners during their learning sessions and the impact of summarized emotional states using mean on the learning flows as shown in Fig. 3

**Table 2:** Description of learning activities dataset.

Number of features	13 numerical features represent learner id, learning activity id, test elapsed time ratio, answer validity ratio, initial learning level, summarized seven classes of ordered facial expressions (angry, disgust, fear, happy, neutral, sad, and surprise), and next learning level. All of these features are significant due to their importance in describing the learning activity and determining the learning path depending on the value of next learning level which is generated by the fuzzy system except both learner id and learning activity id features.
Number of instances	72
• Learning activities dataset using median describes the summarized emotional states using median and its impact on the value of the next learning level of the learner which is generated by a fuzzy system. The description of this dataset is explained in Table 2. Using this dataset we provided visualization for the below.
  a. Aggregated emotional states of five learners across four learning activities for each learner as shown in Fig. 2

**Fig. 2:**
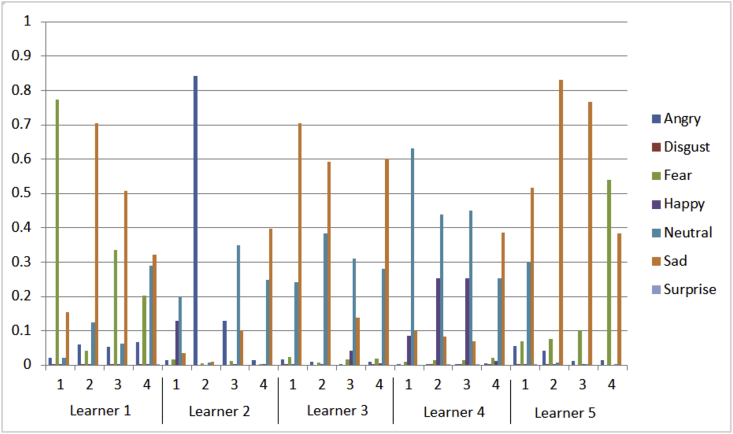
Median of emotional states of five learners across four learning activities.

**Fig. 3:**
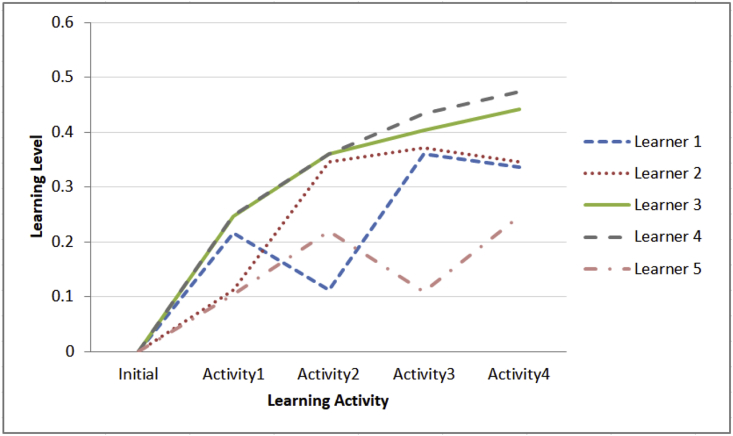
Learning flows of five learners across four learning activities based on the value of next learning level which is generated by fuzzy system depending on summarizing the captured set of emotional states using mean, current learning level, answer validity ratio, and test's elapsed time.
  b. Learning paths for five learners during their learning sessions and the impact of summarized emotional states using median on the learning flows as shown Fig. 4

**Fig. 4:**
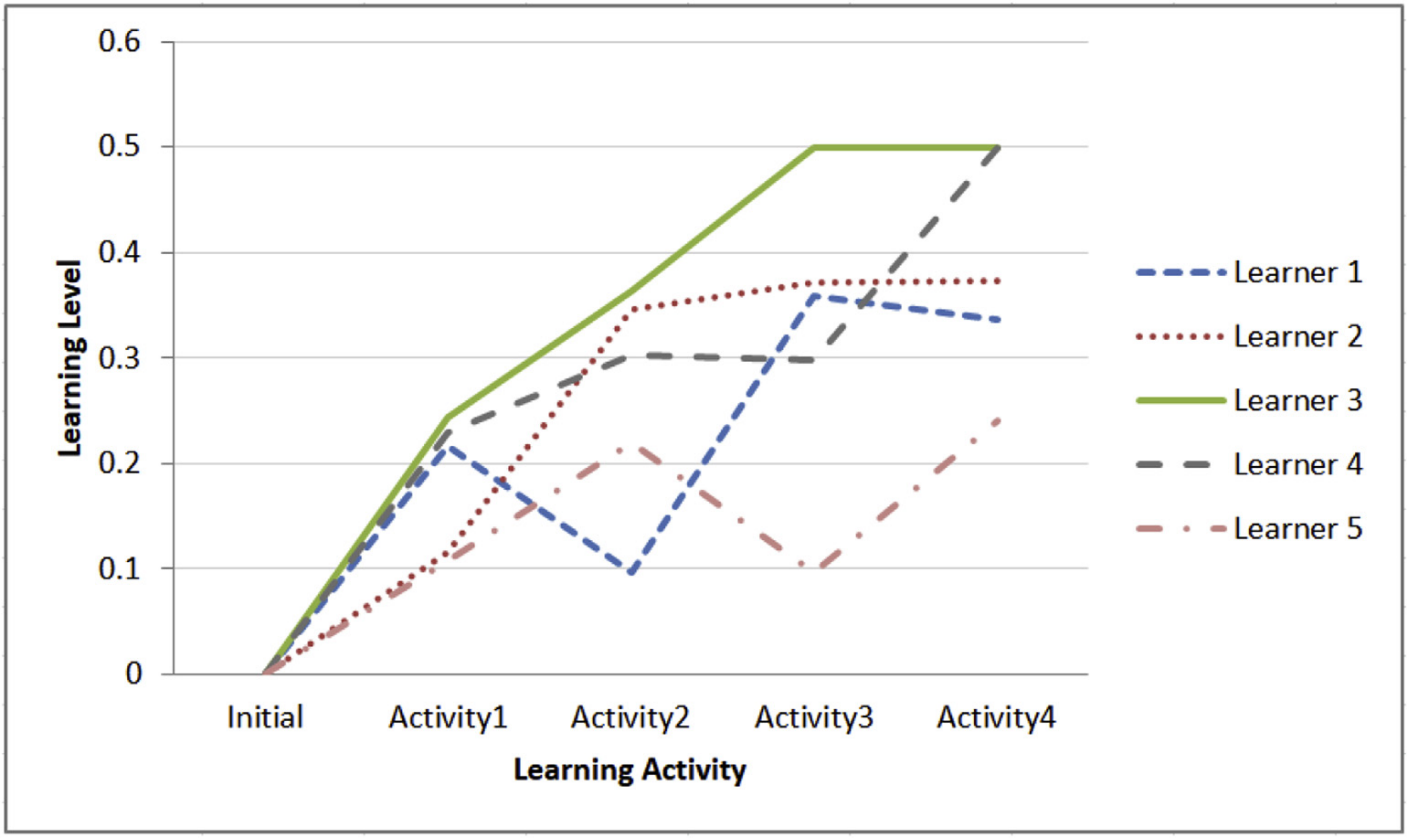
Learning flows of five learners across four learning activities based on the value of next learning level which is generated by fuzzy system depending on summarizing the captured set of emotional states using median, current learning level, answer validity ratio, and test's elapsed time.

## 2 Experimental design, materials, and methods

We developed a dynamic learning environment integrated with real time facial expressions detection model using deep learning to detect the learner's facial expressions and fuzzy system to provide the learning environment with the next learning level based on learner emotional and mental responses.

To test our approach we prepared a test with six levels of difficulties on English language to represent a learning curriculum to a sample group consisted of twelve volunteer people with different ages between 25 and 35 years old, and have different learning background in English language. The sample members have been selected randomly with different ages to verify our approach on different learning levels across different ages, as well to obtain different emotional responses during their interaction with our learning environment. They have participated in the collected data with 72 learning activities represent different learning flows and 1735 data rows of vary emotional states.

We logged all detected facial expressions during the learning sessions except emotional states data records those total value equal to zero in order to provide a consistent and meaningful emotional states data set.

In order to summarize the detected set of emotional states to provide a meaningful crisp value for each facial expression (happy, angry, fear, disgust, surprise, sad, and neutral) we used two different statistical methods. These statistical methods are mean and median to determine which statistical processing on the detected emotional states during the learning sessions is more accurate and realistic to prepare crisp values of emotions for the fuzzy system. The emotional states crisp values were combined with other captured factors (current learning level, test's elapsed time, and answer validity ratio) to represent the crisp inputs to a fuzzy system to provide the next learning level which draws the learning flow.
